# Investigating the Relevance of Nursing Caring Interventions Delivered to Patients with Coronary Artery Disease at a Teaching Hospital in China: A Retrospective Study

**DOI:** 10.7759/cureus.4672

**Published:** 2019-05-15

**Authors:** Fatina B Ramadhani, Yilan Liu, Xue Jing, Ye Qing, Ayoma K Rathnayake, Waheeda Shokat K Kara, Wei Wu

**Affiliations:** 1 Internal Medicine, Union Hospital of Tongji Medical College, Huazhong University of Science and Technology, Wuhan, CHN; 2 Cardiology, Union Hospital of Tongji Medical College, Huazhong University of Science and Technology, Wuhan, CHN; 3 Internal Medicine and Endocrinology, Tongji Medical College, Huazhong University of Science and Technology, Wuhan, CHN; 4 Nursing Psychiatry, Tongji Medical College, Huazhong University of Science and Technology, Wuhan, CHN; 5 Intensive Care, Union Hospital of Tongji Medical College, Huazhong University of Science and Technology, Wuhan, CHN

**Keywords:** cardiology, cardiovascular disease, cardiovascular disease management, cardiovascular disease risk factors, heart health, healthy life style, cardiac rehabilitation, cardiac health counselling, cardiovascular nursing, nursing care

## Abstract

Objective: Coronary artery disease (CAD) remains the leading cause of morbidity and mortality worldwide. Previous systematic reviews and meta-analysis of randomized controlled trials concluded that nursing caring interventions (NCIs) are beneficial for coronary artery patients. However, most of those interventions were conducted in outpatient or home-based settings or through the telephone. Due to its multiple benefits, the authors recommended the application of such interventions to hospitalized coronary artery patients. Currently, little is known on the status of application of such evidence-based interventions in the actual clinical setting for hospitalized coronary artery patients. Similar studies conducted in China were also inadequate. Therefore, this study aimed to investigate the kinds of NCIs delivered to hospitalized coronary artery patients and their consistent relationship with risk factors of CAD found in the clinical records of patients. Results of this study were expected to alert nurses to consider such risk factors when caring for coronary artery patients as well as appraising their caring efforts in improving the patient’s wellbeing for the reduction of morbidity and mortality from a CAD sequel. This report also disseminates some cardiovascular knowledge and health tips to the readers.

Methods: A descriptive, cross-sectional, retrospective design using clinical case notes was employed; the study was undertaken in coronary care wards at the teaching hospital in China from November 2017 to September 2018. A total of 300 coronary artery case notes were randomly selected from 700 eligible cardiovascular patients files by using a simple random technique of simple random numbers through Microsoft office excel sheet. Chi-square (χ^2^) test and multivariate logistic regression analysis for adjusted odds ratio with 95% confidence interval (CI) within its range were used to compare the relationship among independent (patient's demographic and clinical risk factors of CAD) and dependent variables (NCIs implemented to such patients).

Results: A total of 300 coronary artery patients’ case notes were audited with a mean age of 63±11.2 years. Of these 175 (58.3%) were males, 126 (42%) were smokers and 224 (74.7%) were hypertensive. NCIs such as “administer coronary artery medication and their instructions” was mostly delivered to 291 (97%) patients. The delivery of three out of eight gathered NCIs were significantly influenced by three or all of these CAD risk factors (age, smoking, hypertension, and diabetes) (p < 0.05 and/or < 0.01) with an adjusted odds ratio (95% CI) within their significant ranges. Patients with diabetes mellitus were five times more likely to influence the delivery of “administer medication and their instructions” than the rest of patients with coronary artery risk factors (p < 0.01; AOR (95% CI) 5.02(2.059-7.207).

Conclusion: This study reveals that nurses delivered beneficial evidence-based interventions to patients with CAD. The interventions were significantly consistent with age as an unmodifiable risk factor and smoking, hypertension, and diabetes as modifiable risk factors of CAD. However, the management of stress in these patients was low; and since stress may trigger CAD, it should be assessed and managed appropriately.

## Introduction

Coronary artery disease (CAD) remains the leading cause of morbidity and mortality worldwide. CAD also causes about one-third of all deaths in people older than 35 years in western countries while mortality from it is expected to increase in developing countries [1]. It also contributes to emergencies to hospital admissions [2]. In China, the absolute number of cardiovascular (CVD) deaths increased by 46% from 1990 to 2013, with ischemic heart disease such as CAD increasing by 90.9%. The risk of CAD among the Chinese population is increasing as a result of lifestyle changes, urbanization, and increasing number of an aging population [3].

Brief description of coronary artery disease

Coronary artery disease (CAD) arises from occluded coronary artery by deposits of cholesterol, calcium, and abnormal cells [4]. Acute coronary syndrome (ACS) such as unstable angina (UA), myocardial infarction (MI) with or without ST-segment elevation is a group of clinical syndromes caused by rupture of coronary atherosclerotic plaques and secondary thrombosis. It is a manifestation of the long and latent process of atherosclerosis that progressively and asymptomatically begins early in life [5]. Chest pain or discomfort is regarded as the hallmark symptom of ACS, while its absence is regarded as an "atypical" presentation. In this condition, the majority of men with ACS experience chest pain as the primary symptom, and women, who apart from chest pain, often may have upper back, neck, or jaw pain, nausea or fatigue [6]. Apart from a family history of CAD, age, and gender (none-modifiable risk factors), many environmental factors have also been reported to play an important role in the development of CAD, such as tobacco usage, higher body mass index (BMI), dyslipidemia, hypertension, and diabetes mellitus [7].

Over the past two decades, there has been improved care of cardiac patients through pharmacology, modification of cardiac risk factors, education, and application of surgical approaches [8]. The goal of secondary prevention during the acute presentation is to interrupt the thrombotic process so as to restore blood flow for improving myocardial tissue perfusion. Depending on the percentage of coronary artery involved and the nature of blockage, the modern advanced treatment includes percutaneous intervention (PCI) (coronary stents and balloon angioplasty), coronary artery bypass graft (CABG), and pharmacological therapy [9]. 

Patients have also benefited from attending cardiac rehabilitation programs in terms of costs per year and life saved as compared to other well-established preventive and therapeutic interventions in the treatment of CAD such as cholesterol-lowering medication, thrombolysis, coronary angioplasty, surgery or device implantation [10]. However, it was unclear if providers had adhered to instructions and therapy for CAD patients [8]. If not given the right information, patients may lack understanding of their illnesses to their level of health literacy, which would also reduce adherence to medications.

The role of a nurse and nursing caring interventions (NCIs) for coronary artery patients

Among several efforts underway to search for innovative strategies to strengthen the workforce, the major responsibility falls on advanced practice nurses due to an increasing demand for better chronic disease management and improved health care efficiency for patients at a great extent [11]. The nurses' caring role as a counselor and educator during an acute CAD event has been found to be critical for helping patients understand complex therapies involved in treating ACS and developing a plan that fosters medication adherence so as to ensure safety and improved outcomes [12]. It was further added that nurse’s objectives for the discharge of CAD patients included preparation of a patient to return to usual activities. These activities involve understanding the importance of lifestyle and risk factor modification as well as risk reduction like weight management, nutrition, smoking cessation, diet, and physical activity. For more than four decades, nurses have taken on key roles in managing single and multiple risk factors for CAD, including hypertension, diabetes, smoking, and lipids control. Management of these chronic conditions, such as CAD and heart failure, has been done through specialized clinics and programs in primary care, worksites, and cardiac rehabilitation [13-14].

Regardless of the fact that most previous systematic reviews and meta-analysis of randomized controlled trials of nursing interventions for CAD patients found heterogeneity and inconsistency of findings with the outcome set [15-16], still they revealed that NCIs have a beneficial impact on the quality of life of CAD patients [11,15-16]. However, most NCI were for medication, education, and counselling [15] while most trials were conducted at outpatient settings or home or through telephone [16]. Despite that, the authors recommend that such nursing interventions be implemented in the real clinical setting due to the fact that statistically significant findings are not always clinically significant [15]. Currently, not enough evidence exists on the application of the proposed NCIs to hospitalized CAD patients. The existing body of knowledge lacks findings on how NCIs delivered are consistent with CAD patient’s risk characteristics. China is also bereft of such studies. We, therefore, conducted a clinical setting research by tracing clinical records to collect the types of NCIs delivered to CAD patients and weigh out their consistency with regard to patients’ demographic and clinical risks factors for CAD in one of the teaching hospitals in China. Results of this study were expected to alert nurses to consider CAD risk factors when caring for patients as well as appraising their caring efforts in improving the patient’s wellbeing for the reduction of morbidity, frequent attacks, and mortality from a CAD sequel. This report also disseminates some tips to the readers on cardiovascular health which in turn might contribute to the reduction of CAD morbidities.

## Materials and methods

Study design, setting, and population

This research was a descriptive, cross-sectional, clinical records-based retrospective design which was conducted from November 10, 2017 till the required sample was achieved until September 18, 2018. The study was conducted in CAD wards in one of the teaching hospitals in China. Although the university has three International teaching hospitals, the studied hospital had been of interest because it is highly specialized in offering more advanced cardiovascular (CVD) services and surgeries in its region. According to records, the hospital has been successfully performing a total of 3,000 CVD surgeries and 100 heart transplants annually. It also serves other parts of the world apart from China. During our clinical survey, we realized that nurses work in two shifts and at least each nurse can take care of nine to 12 patients daily. Also, nurses in those units have accomplished specialized training in caring for CVD patients.

Inclusion and exclusion criteria

The study targeted discharged cardiovascular (CVD) adult patients (from 18 years old and above) who were diagnosed with CAD within the specified period of time as mentioned above. Recruitment of eligible patients was based on the following criteria: (1) had CAD diagnosis; (2) had age and sex demographic information; (3) had at least one nursing intervention documented in their hospital file. The exception was due to lack of these criteria or other unavoidable hospital logistics. Therefore, all CAD case notes ( In either traditional paper form or electronic or both) which were eligible during the specified period of data collection were randomly selected until the required sample size was achieved.

Sampling and sample size

A single population proportion formula was used to calculate the sample size [17] as follows: n = ( Z)^2^ p (1−p)/d^2 ^ 

n = Minimum sample size required for the study

Z = Value corresponding to the confidence level. For 0.05 confidence level = 1.96

p = Prevalence of stable CAD in previous studies taken as 22.8% [18]

d = Absolute precision (tolerable error) = 0.05

n_0_ = ( Z)^2^ p (1−p)/d^2^ 

n_0_ = (1.96)^2^ 0.23 (1-0.23)/(0.05)^2^

n_0_ = 0.6803 = 272.12/0.0025

n_0_ = 272.12 + ((10%) n_0_) (note: 10%-15% are standard percentages normally added for increasing the sample size)

n_0 _= 272.12 + 27.21 = 299.33

Therefore a sample size of 299.33 was obtained. However, the authors thought that a sample size of 300 was possible and precise for the strength of a single-hospital setting retrospective survey.

A total of 300 eligible CAD files were randomly selected by a simple random technique of random numbers through Microsoft office excel sheet from a pool of about 700 eligible files which were obtained within the specified period of time.

Data collection tool and quality control

Data was collected by using a structured researcher-administered questionnaire. Researchers designed the study questionnaire by utilizing some categorical variables from previous studies especially those which conducted systematic reviews of randomized controlled trials and meta-analysis of nursing interventions for CAD patients. Then “Any other (please specify)” option was purposively utilized in the data collection tool to capture other information apart from those listed by the researchers in the questionnaire. Finally, the questionnaire was divided into three parts (A, B and C): Part A: composed of patient’s demographic and clinical risk variables for CAD such as age, sex, hyperlipidemia, diabetes mellitus, hypertension, smoking, and family history for CAD; based on [19-20] studies. Part B: had information on CAD cardinal signs like cardiac chest pain, back pain and shoulder pain that are reported as common in CAD patients based on sex differences [6]. Part C: composed of nursing interventions for CAD patients; such as education and counseling according to a systematic review of randomized trials of nursing Interventions for secondary prevention in patients with coronary artery disease and heart failure [15]. Nurses roles of administration of CAD medication and their instructions, promotion on diet, lifestyle and risk modification, self-care, and cardiac rehabilitation were proposed in [21-23] studies and systematic review and randomized controlled trials of effective components of nurse-coordinated care to prevent recurrent coronary events [16] and evidence-based analysis of specialized nursing practice for chronic disease management in the primary care setting [11].

Note that clinical symptoms, CAD risky comorbidities, and NCIs variables were dichotomized and given “Yes” or “No” options for each. During data collection, these variables were recorded once if found in the patient’s file without repetitions to avoid some biases of data collectors due to human errors or lack of such repeated information in some files. Additionally, all NCIs found in the records were regarded delivered to patients. To minimize more bias, the absence of such information was regarded as not delivered to the patients. This grouping was just for the convenience of data entry and analysis without any other judgmental meanings. Furthermore, data collection orientation was provided to all researchers to emphasize the uniformity of data collection. Coding of data was done before data collection and cross-checked for consistency and completeness every day.

Validity and reliability of the tool

The tool was composed and structured based on various previous studies' variables and findings. English version questionnaire was translated to fit the local language needs. As part of quality control, the completed tool was reviewed by a panel of nursing research experts for face and content validity and approval to be used for data collection in that region. Additionally, a pretest of 15 (5%) questionnaires (from files before November 10, 2017) was conducted. An analysis of the pretest results guided appropriate modifications of the tool before the actual data collection for the full major study.

Data analysis

Data was entered, cleaned, proofread, coded and analyzed using the Statistical Package for Social Sciences software (SPSS), version 23 (IBM Corp., Armonk, NY). Frequency tables were displayed to present the summary of categorical variables while for continuous variables, means, standard deviations (SD) and ranges were used to summarize the information. Univariate analysis through chi-square test (x2) was used for comparing the delivery of NCIs (dependent variables) due to independent variables (patients' demographic and clinical CAD risky characteristics). A multivariate logistic regression model was used for adjusting confounding variables like the patient’s chest, back, and abdominal pain symptoms. During analysis, all variables were treated as categorical except for the computation of means and SD for age variables. P-value in a downward direction from 0.05 and adjusted odds ratios (AOR) with 95% confidence interval (CI) ranges were stated as significant findings.

Ethical considerations

Permission to conduct this study was granted by the research ethical committee of Tongji Medical College of Huazhong University of Science and Technology (HUST), China. However, this research didn’t require physical contact with the participant as it used the patient’s hospital records. Furthermore, researchers handled the CAD patient’s clinical information with very high confidentiality. Authors also used unique identifiers for eligible CAD case files so as to increase the privacy of the patients’ documents.

## Results

Demographics and clinical characteristics of patients

A total of 300 CAD patients’ case notes were audited. Their age ranged from 33-99 years old with mean and standard deviation (SD) of 63±11.2 years old. Table 1 below shows demographic characteristics which are among CAD risk factors. Almost one third (95; 31.7%) fell under the age group of 60-69 years and more than half were males 175 (58.3%). Aging and sex are both unmodifiable CAD risk factors. However, younger ones also look affected with CAD whereby 41.3% were below 60 years. Cigarette smoking as a modifiable CAD risk factor appeared to nearly half 126 (42.0%) of the group which indicates that most of them had been active smokers.

**Table 1:** Social-demographic risk characteristics of CAD patients, n=300 CAD: coronary artery disease

Variables		n (%)
Sex	Male	175 (58.3)
	Female	12 (41.7)
Age	30-59	124 (41.3)
	60-89	173 (57.7)
	90+	3 (1.0)
Cigarette smoking	Yes	126 (42.0)
	No	174 (58.0)
CAD family history	Yes	107 (35.7)
	No	193 (64.3)

Table 2 below shows clinical characteristics in terms of risky comorbidities and cardinal signs and symptoms of CAD. More than half of the patients (191; 63.7%) of patients presented with cardiac chest pain. Cardiac chest pain is a major chief complaint and cardinal sign of CAD. Additionally, hypertension as comorbidity for CAD existed in almost three-quarter of patients (224; 74.7%) followed by diabetes mellitus (75; 25.0%).

**Table 2:** Risky comorbidities, cardinal signs/symptoms of CAD patients, n=300 CAD: coronary artery disease

Variables		Found	Not found
		n (%)	n (%)
Signs/symptoms	Cardiac chest pain	191 (63.7)	109 (36.3)
	Back pain	48 (16.0)	252 (84.0)
	Abdominal pain	33 (11.0)	267 (89.0)
Comorbidities	Hypertension	224 (74.7)	76 (25.3)
	Diabetes mellitus	75 (25.0)	225 (75.0)
	Hyperlipidemia	46 (15.3)	254 (84.7)

Nursing caring interventions delivered to patients

Table 3 below lists the type of NCIs delivered to CAD patients. Note that during data collection authors did not consider how repeatedly one NCI was documented to each patient due to insufficient information of such clinical records. Therefore, the authors picked single recording of NCI from each patient case note. Finally, eight kinds of NCIs were identified. Of these “administer CAD medication and their instructions” was delivered to the most number of patients (291; 97%) while “counsel to cope with stress” was the least delivered (60; 20.0%).

**Table 3:** Nursing caring interventions delivered as found documented in the patient’s records, n=300 ABG: arterial blood gas; BP: blood pressure; BG: blood glucose; CAD: coronary artery disease; ECG: electrocardiogram.

Nursing caring interventions	Found	Not found
	n (%)	n (%)
Assess to grade pain severity on 1-10 scale	206 (68.7)	94 (31.3)
Identify pain precipitating activities	210 (70.0)	90 (30)
Administer CAD medication and their instructions	291 (97.0)	9 (3.0)
Educate on cardiac rehabilitation skills	285 (95.0)	15 (5.0)
Counsel on diet and life style modification	289 (96.3)	11 (3.7)
Offer human caring (reassurance, self-care, family counseling)	284 (94.7)	16 (5.3)
Monitor vital signs like (BP, BG, ECG, hemodynamics, ABG and lipids)	235 (78.3)	65 (21.7)
Counsel to cope with stress	60 (20.0)	240 (80.0)

Statistical test results

The table below shows univariate analysis results through the chi-square test (χ2) for comparing nursing interventions delivered as dependent variables, and the patient’s CAD risks factors as independent variables. Most NCIs showed statistically significant different results with CAD risk factors of patients. Therefore there is evidence that NCIs were delivered due to one or more CAD risk factors of patients (p < 0.05 and/or < 0.001). The delivery of two NCIs like “administer CAD medication and their instructions” and “offer human caring (reassurance, self-care, family counseling)” was significantly related to all risk variables (p < 0.05 < 0.01). Therefore, there is evidence that the delivery of NCIs has been considering the risk characteristics of CAD patients (Table 4).

**Table 4:** χ2 test for comparing delivered nursing caring interventions with the patient’s CAD risk factors, n=300 *p < 0.05; **p < 0.01. ABG: arterial blood gas; BP: blood pressure; BG: blood glucose; CAD: coronary artery disease; ECG: electrocardiogram; HT: hypertension; DM: diabetes mellitus; HL: hyperlipidemia.

Nursing Caring interventions		n (%)	Age	Sex	Smoking	CAD-Family	HT	DM	HL
Assess to grade pain severity on 1-10 scale	206 (68.7)		0.02	0.11	0.02	0.62	0.01*	0.06	0.05
Identify pain precipitating activities	210 (70.0)		0.02	0.37	0.02	0.62	0.15	0.01*	0.02
Administer CAD medication and their instructions	291 (97.0)		0.01**	0.04	0.03	0.01	0.04	0.02	0.04
Educate on cardiac rehabilitation skills	285 (95.0)		0.02	0.78	0.03	0.08	0.01*	0.01*	0.07
Counsel on diet and life style modification	289 (96.3)		0.34	0.42	0.03	0.65	0.01*	0.01**	0.17
Offer human caring (reassurance, self-care, family counseling)	284 (94.7)		0.03	0.01*	0.01*	0.02	0.01**	0.001*	0.03
Monitor vital signs (like BP, BG, ECG, hemodynamics, ABG and lipids)	235 (78.3)		0.08	0.32	0.04	0.90	0.04	0.16	0.71
Counsel to cope with stress	60 (20.0)		0.47	0.10	0.62	0.88	0.32	0.01	0.63

The table below shows the multivariate logistic regression model for adjusting confounding variables like pain symptoms and patients risk variables with the comparison of NCIs delivered with patients' CAD risks factors. Results show strong evidence that delivery of three NCIs significantly matched with either age and/or smoking, hypertension and diabetes; CAD risky variables (p < 0.01; AOR (95% CI). Patients with diabetes were five times more likely to influence the delivery of “administer CAD medication and their instructions” than the rest (p < 0.01; AOR (95% CI) 5.02 (2.059-7.207) (Table 5). This gives strong evidence that delivery of some NCIs was consistently relevant with some risk factors of CAD patients.

**Table 5:** Multivariate logistic regression analysis for adjusting the comparison of NCIs delivered with patient’s CAD risk factors, n=300 *p < 0.05; **p < 0.01. CAD: coronary artery disease; AOR: adjusted odds ratio; CI: confidence interval; NCIs: nursing caring interventions.

Nursing interventions	Age	Smoking	Hypertension	Diabetes mellitus
	AOR (95% CI)	AOR (95% CI)	AOR (95% CI)	AOR (95% CI)
Administer CAD medication and their instructions	2.603 (2.341-3.103)*	1.009 (1.002-4.006)*	1.202 (1.107-3.824)*	5.02 (2.059-7.207)**
Educate on cardiac rehabilitation skills	0.008 (1.008-2.313)	0.106 (0.102-2.134)*	1.439 (1.206-4.128)*	0.808 (0.508-2.096)**
Counsel on diet and life style modification	0.095 (2.023-5.360)	1.835 (1.325-5.861)*	1.429 (1.111-1.793)**	1.095 (1.001-5.397)*

## Discussion

This study intended to investigate if evidence-based beneficial nursing caring interventions (NCIs) for CAD patients are applied in the real clinical setting and if they are consistent with the demographic and clinical risky characteristics of CAD patients. Nearly half (41.2%) of the patients in this study were below 50 years with mean age and SD of (63±11.2) while the range was between 33 to 99 years. The proportion of male patients was higher (58%) although relatively close to female patients. This means that, as of recent times, the disease affects both genders and even younger adults. Similar findings reported that in the past, CAD was a disease of older ages but nowadays younger people are also affected [24]. However, studies revealed that age, sex, and history of CAD in a family are all un-modifiable risk factors of CAD. In this study, most of our patients also presented with modifiable risk factors like hypertension and cigarette smoking history. All these are among the typical and traditional risk factors found in CAD patients as described in previous studies [19-20].

On the other hand, the results of this study found more promising evidence-based and beneficial NCIs to be delivered for hospitalized CAD patients. A total of eight evidence-based NCIs were delivered to hospitalized CAD patients. “medication administration with their instructions” took a leading proportion (97%) while management of patient’s stress did not receive enough attention and hence, achieved a very small percentage (20%) of delivery. This big difference might be due to less cumulative documentation of social psychological intervention event in most clinical settings. Despite that discrepancy, NCIs gathered in this study outweigh 65% of secondary nursing interventions for CAD patients found in other studies which were all for educational and behavioral counseling. Still, in their findings, more than half of the interventions were not significant and not consistent with the intended outcome measures as well [15]. On the contrary, NCIs gathered in the current study were found relevant and significantly consistent with traditionally known CAD risk factors of patients [20]. In this study, the nursing CAD medication administration role paralleled with their instructions, promotion of heart-healthy diet, life style modification and education on cardiac rehabilitation have shown strong evidence that they were delivered with consideration of patient’s CAD risky status. These interventions have been confirmed beneficial for CAD patient’s wellbeing [21]. This indicates that recently there have been extra efforts in cardiovascular nursing to meet the needs of hospitalized CAD patients. Perhaps this is contributed by on-the-job training of nurses on specialty care as well as advancement and emerging of various nursing specialties such as cardiovascular nursing that allows more focus on patient’s risk factors for reducing CAD morbidity and adverse outcomes. Other studies revealed that chronic management of CVD will be achieved only if patients understand and follow prescribed treatment regimens [25-26].

On the other hand, the current study is confident to reveal that the provision of NCIs like education on cardiac rehabilitation skills and counselling on diet and life style modification was consistent with patient’s modifiable CAD risk factors like smoking and comorbid conditions like hypertension and diabetes. We think this is an impressive observation that, nurses considered delivery of such intervention when caring for such patients. Our findings are supported by the evidence whereby such interventions have been delivered because they are beneficial for cardiac stability and minimizing of frequent CAD attacks [24]. Education regarding lifestyle factors has been found as an essential part of the discharge process [12,27]. Several studies on cardiac rehabilitation for CAD patients suggested that, it is imperative that patients receive proper medical management of coronary risk factors and support for the adoption of a healthy lifestyle [28]. Furthermore, patients deserve special attention to restore their quality of life, to maintain or improve functional capacity following interventions of acute events [10]. Therefore in achieving that they require counselling to prevent event recurrence, by adhesion to a medication plan and adoption of a healthy lifestyle. Interventions like “education on diet and life style modification” have been used in promoting heart health behaviors. Similar heart health promotion behaviors have been associated with cardiac rehabilitation programs (CRP) in reducing the risk of coronary events and coronary death [28]. Cardiac rehabilitation programs (CRP) improve risk factors, medication adherence to secondary preventive therapies, exercise capacity, and survival after percutaneous coronary interventions (PCI) and coronary artery bypass grafting (CABG) surgery [23].

By considering the importance of medication administration and their proper instruction in improving CAD patient’s wellbeing, we give credits to all nurses who took care of the studied patients for their great job basing on our findings. However, this NCI “councel the patient to cope with stress” we disregard as it has been delivered in the lowest due to its equally critical importance and benefits for CAD patients. Our worry is supported by the fact that psychological factors such as depression and stress have been listed as among the underlying risk factors for CAD [29]. Regardless of the fact that nurses can face time constraint and inadequate staffing, nursing practices can still be developed with technology that improves the physical environment to provide CAD patients with the opportunity to feel better about themselves. With the support of the evidence, the authors recommend that stress is necessary to be assessed and managed as it may trigger CAD attack, and hence, hinder the patient’s wellbeing.

Limitation of the study

Our study might have limited generalizability due to the use of a single center; hence, it could be appropriate for the population attending the teaching hospital under study. Nevertheless, we believe that the large data set used increases the power of the study and minimizes the single-center limitation. However, interpretation of our findings should be done with precaution as this was a study from a highly specialized teaching hospital and among the best ones in providing cardiovascular services in China. Additionally, this study has also found scarce evidence of similar kinds of studies. Hence, further research is urgently needed on the related subject through, for example, a multicenter study employing either similar or different methods may help in elucidating this relationship and possible beneficial impacts for CAD patients. Furthermore, evaluation of the patient’s knowledge and experiences following the reception of educational and counselling components on cardiac rehabilitation and related ill health behaviors is crucial. Nurses can also explore their experiences of caring for CAD patients for further improvement of care and patients well-being. Lastly, research is needed to get the consistency of nursing interventions' impact with the patient’s clinical end-point outcomes such as mortality/recovery rate, length of stay, and satisfaction of nursing caring behaviors for further appraisal of nursing efforts in caring for CAD patients.

## Conclusions

This study reveals that modifiable risk factors like hypertension increasingly affect CAD patients. However, the study delivers valuable insight that, nurses have been delivering beneficial evidence-based nursing caring interventions to hospitalized CAD patients which significantly matched with patients' risk factors of coronary artery illness. Moreover, following adjusted analysis of controlling confounding variables, significant different findings were seen in only three nursing interventions “administer CAD medication and their instructions, educate on cardiac rehabilitation skills, and counsel on diet and life style modification”. These were significantly consistent with either age and/or smoking, hypertension, and diabetes CAD risk variables, whereby diabetes illness led to this influence. However, we found a small percentage of documentation of NCI "councel to cope with stress"; hence, nurses have to improve their care and documentation of this important psychosocial aspect for CAD patients. For example, based on the nursing assessment done, nurses can document potential aspects of stress and the specific stress-relieving advice given to a particular patient. Furthermore, research is needed to get the consistency of nursing caring interventions with patient’s end clinical outcomes such as recovery, length of stay, and satisfaction with caring nursing behavior for further appraisal of nursing efforts in caring for CAD patients.

## References

[1] Sanchis-Gomar F, Perez-Quilis C, Leischik R, Lucia A (2016). Epidemiology of coronary heart disease and acute coronary syndrome. Ann Transl Med.

[2] Sanchis-Gomar F, Perez-Quilis C, Leischik R, Lucia A (2019). What Is coronary artery disease?. Ann Transl Med.

[3] Chen WW, Gao RL, Liu LS (2017). China cardiovascular diseases report 2015: a summary. J Geriatr Cardiol.

[4] Andrikopoulos G, Terentes-Printzios D, Tzeis S (2016). Epidemiological characteristics, management and early outcomes of acute coronary syndromes in Greece: The Phaenthon study. Hell J Cardiol.

[5] Singh V, Kumari G, Chhajer B, Dahiya S (2017). Significant improvement in clinical symptoms and quality of life of coronary heart disease with diabetes mellitus patients treated with EECP therapy. Int J Adv Sci Eng Technol.

[6] Canto GJ, Goldberg JR, Hand M, Bonow RO, Sopko G, Pepine CJ, Long T (2007). Symptom presentation of women with acute coronary syndromes: myth vs reality. Arch Intern Med.

[7] Yoon SS, Dillon CF, Illoh K, Carroll M (2016). Trends in the prevalence of coronary heart disease in the U.S. Am J Prev Med.

[8] Bortnick AE, Epps KC, Selzer F (2014). Five-year follow-up of patients treated for coronary artery disease in the face of an increasing burden of co-morbidity and disease complexity. Am J Cardiol.

[9] Brilakis ES, Rao SV, Banerjee S (2011). Percutaneous coronary intervention in native arteries versus bypass grafts in prior coronary artery bypass grafting patients. J Cardiovasc Interv.

[10] Piepoli MF, Hoes AW, Agewall S (2016). 2016 European Guidelines on cardiovascular disease prevention in clinical practice. Eur Heart J.

[11] Health Quality Ontario (2013). Specialized nursing practice for chronic disease management in the primary care setting: an evidence-based analysis. Ont Health Technol Assess Ser.

[12] Berra K, Fletcher JB, Handberg E (2011). Antiplatelet therapy in acute coronary syndromes implications for nursing practice. J Cardiovasc Nurs.

[13] Wal P, Wal A, Rai AK, Nair VR, Pandey U (2013). Management of coronary artery disease in a tertiary care hospital. J Basic Clin Pharm.

[14] Yang Q, Wang Y, Liu J (2017). Invasive management strategies and antithrombotic treatments in patients with non-ST-segment-elevation acute coronary syndrome in China. Circ Cardiovasc Interv.

[15] Allen JK, Dennison CR (2010). Randomized trials of nursing interventions for secondary prevention in patients with coronary artery disease and heart failure systematic review. J Cardiovasc Nurs.

[16] Snaterse M, Dobber J, Jepma P (2016). Effective components of nurse-coordinated care to prevent recurrent coronary events: a systematic review and meta-analysis. Heart.

[17] Jember A, Hailu M, Messele A, Demeke T, Hassen M (2018). Proportion of medication error reporting and associated factors among nurses: a cross sectional study. BMC Nurs.

[18] Yang S, Zhou Y, Zhao Y (2017). The serum anion gap is associated with disease severity and all-cause mortality in coronary artery disease. J Geriatr Cardiol.

[19] Khalili D, Sheikholeslami FH, Bakhtiyari M, Azizi F, Momenan AA, Hadaegh F (2014). The incidence of coronary heart disease and the population attributable fraction of its risk factors in Tehran: a 10-year population-based cohort study. PLoS One.

[20] Smith SCJ, Benjamin EJ, Bonow RO (2011). AHA/ACCF secondary prevention and risk reduction therapy for patients with coronary and other atherosclerotic vascular disease: 2011 update. J Am Coll Cardiol.

[21] Hayman LL, Berra K, Fletcher BJ, Houston Miller N (2015). The role of nurses in promoting cardiovascular health worldwide. J Am Coll Cardiol.

[22] Berra K, Fletcher BJ, Hayman LL, Miller NH (2011). Global cardiovascular disease prevention: a call to action for nursing the global burden of cardiovascular disease. Eur J Cardiovasc Nurs.

[23] Leon AS, Franklin BA, Costa F (2005). Cardiac rehabilitation and secondary prevention of coronary heart disease. Circulation.

[24] Piepoli MF, Corrà U, Benzer W (2010). Secondary prevention through cardiac rehabilitation: from knowledge to implementation. A position paper from the Cardiac Rehabilitation Section of the European Association of Cardiovascular Prevention and Rehabilitation. Eur J Cardiovasc Prev Rehabil.

[25] Simoens S, Sinnaeve PR (2014). Patient co-payment and adherence to statins: a review and case studies. Cardiovasc Drugs Ther.

[26] Azzolin KD, Lemos DM, Lucena AD, Rabelo-Silva ER (2015). Home-based nursing interventions improve knowledge of disease and management in patients with heart failure. Rev Lat Am Enfermagem.

[27] Krantz MJ, Baker WA, Estacio RO, Haynes DK, Mehler PS, Fonarow GC, Long CS (2007). Comprehensive coronary artery disease care in a safety-net hospital: Results of get with the guidelines quality improvement initiative. J Manag Care Pharm.

[28] Payne FE, Boineau JP (1980). Cardiac rehabilitation. Am Fam Physician.

[29] Kazemian F, Jalali SF, Hajian-Tilaki K, Arzani A, Amin K (2018). Underlying risk factors and their relationship with extent of coronary vessel involvement in patients undergoing coronary angiography in North of Iran. Caspian J Intern Med.

